# Transcriptional Landscapes of Divergent Sporophyte Development in Two Mosses, *Physcomitrium (Physcomitrella) patens* and *Funaria hygrometrica*

**DOI:** 10.3389/fpls.2020.00747

**Published:** 2020-06-10

**Authors:** Alexander Kirbis, Manuel Waller, Mariana Ricca, Zoe Bont, Anna Neubauer, Bernard Goffinet, Péter Szövényi

**Affiliations:** ^1^Department of Systematic and Evolutionary Botany, University of Zurich, Zurich and Zurich-Basel Plant Science Center, Zurich, Switzerland; ^2^Department for BioMedical Research (DBMR), University of Bern, Bern, Switzerland; ^3^Institute of Plant Sciences, University of Bern, Bern, Switzerland; ^4^Department of Ecology and Evolutionary Biology, University of Connecticut, Storrs, CT, United States

**Keywords:** transcriptomics, sporophyte development, mosses, Funariaceae, RNAseq

## Abstract

Understanding the molecular basis of morphological shifts is a fundamental question of evolutionary biology. New morphologies may arise through the birth/death of genes (gene gain/loss) or by reutilizing existing gene sets. Yet, the relative contribution of these two processes to radical morphological shifts is still poorly understood. Here, we use the model system of two mosses, *Funaria hygrometrica* and *Physcomitrium (Physcomitrella) patens*, to investigate the molecular mechanisms underlying contrasting sporophyte architectures. We used comparative analysis of time-series expression data for four stages of sporophyte development in both species to address this question in detail. We found that large-scale differences in sporophytic architecture are mainly governed by orthologous (i.e., shared) genes frequently experiencing temporal gene expression shifts between the two species. While the absolute number of species-specific genes expressed during sporophyte development is somewhat smaller, we observed a significant increase of their proportion in preferentially sporophyte expressed genes, suggesting a fundamental role in the sporophyte phase. However, further functional studies are necessary to determine their contribution to diverging sporophyte morphologies. Our results add to the growing set of studies suggesting that radical changes in morphology may rely on the heterochronic expression of conserved regulators.

## Introduction

The genome is constantly reshaped by diverse types of mutations providing raw material for evolution to work with. Genomic changes can ultimately lead to new phenotypes possessing a novel set of morphological characters potentially enabling adaptation to new environmental conditions (Orr, 2005). Various molecular mechanisms may underlie the origin of morphological novelties including (i) the rise of novel genes (de novo evolution and introgression of genes) (Chen et al., 2013; Schlötterer, 2015; Li et al., 2016; Zhang et al., 2019), (ii) the utilization of existing genes for new functions (Davidson, 2010; Pires and Dolan, 2012; Das Gupta and Tsiantis, 2018; Bowman et al., 2019), (iii) and the loss of genes or gene function (Albalat and Cañestro, 2016; Xu et al., 2019). Nevertheless, in reality molecular mechanisms underlying the origin of morphological novelties are more complex and can be best described by various combinations of these three basic scenarios.

Although the primary molecular mechanisms contributing to novel phenotypes are well-documented, their relative importance is poorly understood and difficult to predict (True and Carroll, 2002; Khalturin et al., 2009; Kaessmann, 2010; Wagner and Lynch, 2010). For instance, *de novo* evolution of genes is thought to have boosted the diversification of plants by enabling the evolution of new key characters both at the molecular and macromorphological levels (Furumizu et al., 2015; Soltis and Soltis, 2016; Jill Harrison, 2017; Van de Peer et al., 2017; Clark and Donoghue, 2018; Landis et al., 2018; Whitewoods et al., 2018; One Thousand Plant Transcriptomes Initiative, 2019; Bowles et al., 2020). Similarly, new genes acquired by horizontal gene transfer or introgression/hybridization seem to have also been crucial in the evolution of key morphological features (Suarez-Gonzalez et al., 2018; Cheng et al., 2019; Wickell and Li, 2020). In contrast, the evolution of many key phenotypic characters has taken another path by co-opting existing genes or complete regulatory networks to create new morphological features (Rast-Somssich et al., 2015; Rebeiz and Tsiantis, 2017). Often, temporal shifts in the expression of conserved regulatory modules (e.g., heterochronic expression) is sufficient to give rise to new morphological innovations both in plant and animal systems (Geuten and Coenen, 2013; Buendía-Monreal and Gillmor, 2018). For instance, heterochronic expression of some key genes are major determinants of organ size and number in *Arabidopsis* (Sun et al., 2014). Finally, evidence is mounting that new phenotypes can also be acquired by loss of genes or gene functions. For instance, rapid evolution of new phenotypes seems to have proceeded by compromising gene function both in animals and plants (Olson, 1999; Nachman et al., 2003; Gujas et al., 2012; MacArthur et al., 2012; Nadeau et al., 2016; Sun et al., 2018). Therefore, the underlying molecular processes leading to the evolution of new morphological structures are diverse and it is currently unclear why evolution of particular phenotypes would follow one or the other trajectory (see i-iii above). Furthermore, whether evolutionary trajectories are canalized by various currently poorly known constraints or the prevalence of one trajectory is rather determined by random chance is highly debated (Galis et al., 2018). Revealing the molecular processes underlying phenotypic changes in a diverse set of model systems may help to discover key commonalities of the evolutionary process and assess how gene gain/loss and co-option of existing genes for new functions may contribute to morphological evolution.

Here, we use a model system of two species from a single family of mosses, *Physcomitrium (Physcomitrella) patens* (hereafter referred to as *P. patens*, see in Medina et al., 2019) and *Funaria hygrometrica*, to begin investigating the molecular mechanisms shaping the evolution of their highly distinct sporophyte morphologies. In contrast to flowering plants, the moss life cycle possesses a dominant haploid gametophyte (1n) (consisting of the filamentous protonema and leafy shoot-like gametophores) alternating with an unbranched diploid sporophyte (2n) phase. The diploid sporophyte phase is multicellular, photosynthetic although nutritionally dependent on the maternal gametophyte to which it is permanently attached (Jonathan Shaw and Goffinet, 2000). The primary function of the sporophyte is to produce spores and control their dispersal, and therefore the sporophyte is likely under severe selection (Haig, 2013).

**FIGURE 1:**
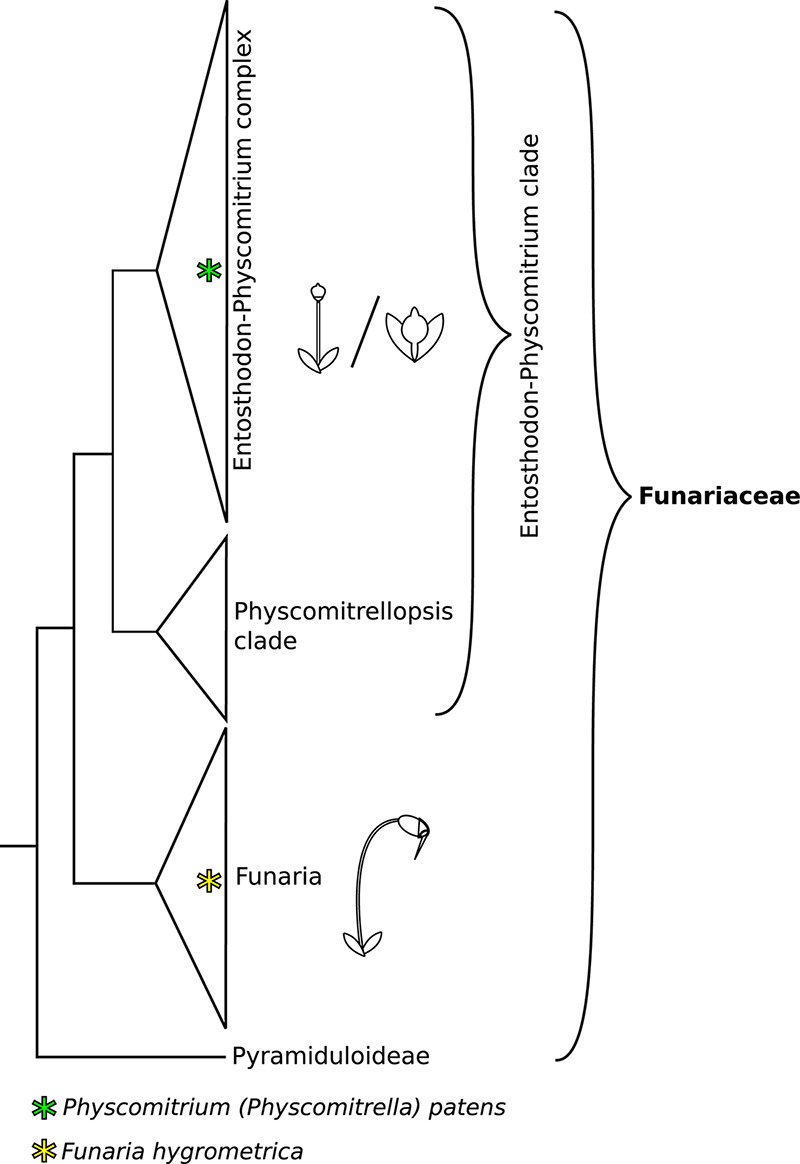
Phylogeny backbone of the moss family Funariaceae, redrawn from Medina et al. (2018, 2019). Species of the *Funaria* clade show a complex sporophyte phenotype, characterized by an elongated seta and structures aiding controlled spore dispersal (operculum, revoluble annulus, peristome teeth). The *Entosthodon*-*Physcomitrium* (EP) clade is mainly comprised of species with an intermediate sporophyte phenotype, lacking one or several structures for improved control of spore dispersal and displaying an overall reduced size. Within the EP clade severe reduction of sporophyte complexity occurred at least four times independently. These highly reduced sporophytes are short and lack specialized structures aiding spore dispersal, mainly forming simple, indehiscent capsules.

**FIGURE 2:**
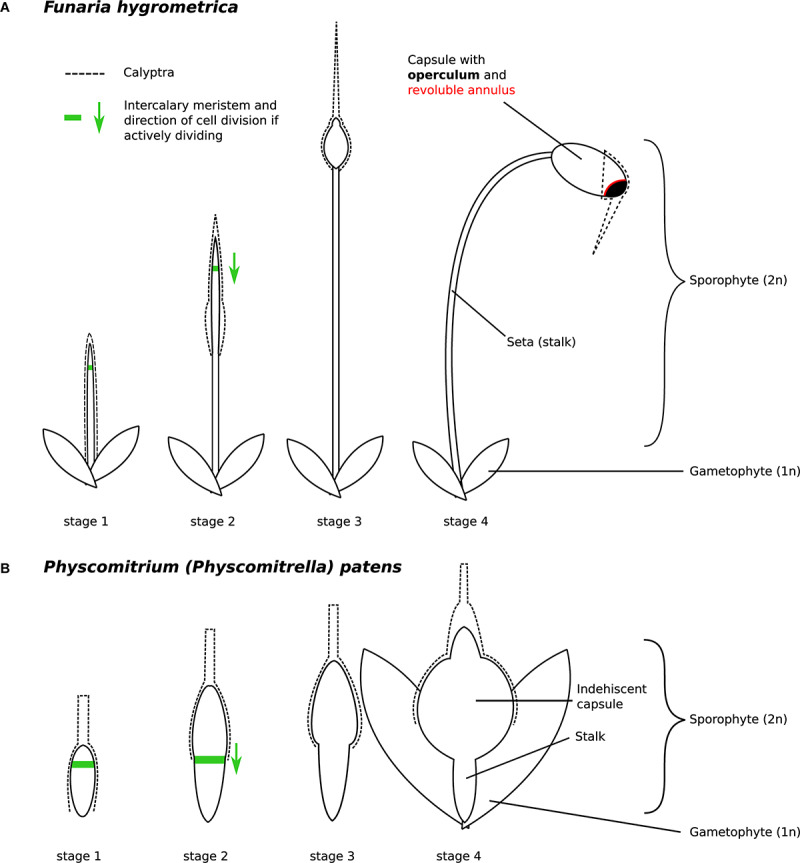
Developmental stages of **(A)** *Funaria hygrometrica* and **(B)** *Physcomitrium patens* sporophytes investigated in this study. The stages were characterized as follows: stage 1 – sporophyte emerges from gametophytic tissue, stage 2 – elongation of the seta occurs, stage 3 – sporophyte reaches its final height, capsule begins to swell, stage 4 – capsule has swollen to its final size and all specialized structures are established but meiosis has not occurred yet. Presence of the green arrow indicates that the intercalary meristem (green line) is likely active and shows the direction of cell division. The *F. hygrometrica* sporophyte reaches a final size of 5 cm, the *P. patens* sporophyte grows to a size of 1 mm. The calyptra (dashed line) is developed from the archegonium and as such, is not part of the sporophyte.

The moss family Funariaceae comprises about 300 species, displaying a relatively uniform gametophyte morphology, but highly variable sporophyte stature varying in size from about a millimeter to 5 cm and in complexity from a sessile sporangium lacking a differentiated mode of dehiscence to a long stalked capsule bearing highly specialized structures for controlled spore dispersal (Medina et al., 2018, 2019). Phylogenomic analyses revealed that the subfamily Funarioideae comprises the monophyletic *Funaria* and its sister lineage, the *Entosthodon-Physcomitrium* (EP) complex (Figure 1) with an estimated divergence time of 60 million years (95% CI: 35–70 million years ago; Medina et al., 2018). *Funaria* is characterized by an architecturally rather complex sporophyte, with an elongated seta, asymmetric capsule, dehiscing via a revoluble double annulus revealing a double peristome regulating spore dispersal (Fife, 1982; Liu et al., 2012; Medina et al., 2018). By contrast, the sporophyte of the E-P complex is more variable, spanning a gradient of architectural complexity extending to the simple sporophyte of *P. patens* whose sporophyte is composed of a short seta and a small spherical capsule lacking differentiated structures associated with spore dispersal (Figures 1, 2). Given the resolution of *P. patens* within a grade of *Entosthodon* and *Physcomitrium* species, all with more complex sporophytes, it is assumed that the sporophyte morphology of *P. patens* arose through reduction (Liu et al., 2012; Medina et al., 2018). Ontogenetic transformations of the sporophyte are correlated or at least followed by changes in the development of the calyptra, the protective maternal gametophytic tissue covering the apex of the developing sporophyte (Budke and Goffinet, 2016). Reduction of the diploid generation occurred multiple times in the complex, giving rise to several species with a *Physcomitrium* phenotype (Medina et al., 2019). Whether such transformations were triggered by similar genetic processes is not known, and in fact the molecular mechanisms underlying sporophyte development in mosses, in particular that of contrasting sporophyte morphologies in the Funariaceae, are poorly understood (Goffinet and Buck, 2013).

We used comparative gene expression and genome analysis to begin investigating the molecular changes that potentially contribute to the evolution of the contrasting sporophyte morphology in Funariaceae. To do so, we generated time-series gene expression data for four comparable stages of sporophyte development in two funarioid mosses, *F. hygrometrica* (complex sporophyte) and *P. patens* (simple sporophyte), representing the two extremes of sporophyte complexity in the family. We contrasted gene expression in the two species to test whether shifts in sporophyte complexity were associated with changes in expression of shared genes or with changes in gene content, e.g., gene loss/gain. Our analyses suggest that heterochronic expression of conserved sets of developmental genes govern the development of sporophytes with contrasting complexities, although the contribution of gene gain/loss is also considerable. Our study adds to the growing set of observations that reorganization of highly conserved regulatory networks is a critical mechanism underlying major morphological shifts in evolution (Rosin and Kramer, 2009; Zhao et al., 2016; Das Gupta and Tsiantis, 2018; Bowman et al., 2019).

## Materials and Methods

### Extraction of RNA From Sporophyte Tissue of *F. hygrometrica* and *P. patens*

The *F. hygrometrica* isolate used in this study was established from a single spore of a sporophyte collected around the city of Sankt Gallen, Switzerland. Gametophores were grown and vegetatively propagated in petri dishes on KNOP medium (Reski and Abel, 1985). To obtain sporophytes, gametophores were coarsely ground, and fragments spread on sterilized soil. Cultures were regularly sprayed with water to facilitate fertilization and sporophyte development was checked weekly. We collected sporophytes of *F. hygrometrica* corresponding to the four developmental stages described in Budke et al. (2012) and depicted in Figure 2A. We also sampled *P. patens* sporophytes with comparable developmental stages as shown in Figure 2B. For RNA-sequencing, we sampled sporophytes using forceps and scissors. We also used spores from freshly collected sporophytes to obtain plant material for various developmental stages of the gametophyte phase. We spread spores of surface sterilized sporophytes onto Knop medium overlaid with cellophane and collected germinating spores after 3 days (germinating spores 3 days) and 2 weeks (protonema 2 weeks). Young gametophores (young buds) emerging after 3 weeks were also collected. We immediately submerged collected sporophytes and gametophyte tissues into RNAlater^®^ and stored them at 4°C until RNA extraction. RNA was extracted using the Spectrum^TM^ Plant Total RNA Kit from Sigma-Aldrich. The calyptra was removed from all sporophytic tissues prior RNA extraction. Poly-A RNA of three biological replicates were sequenced with paired-end or single-end sequencing on a HiSeq 2500 or 4000 machine (see Supplementary Table S1).

### Draft Genome Sequence of *F. hygrometrica*

We extracted DNA using a modified CTAB protocol (Porebski et al., 1997) from axenically grown gametophytes of the very same *F. hygrometrica* isolate we used for the RNA-seq experiment. We first prepared 454 libraries, which were sequenced with the titanium technology on a 454 FLX Roche machine at the Functional Genomic Center Zurich (FGCZ). We prepared short-insert DNA libraries and 3 and 5 kb jumping libraries using the Illumina DNA-seq and mate-pair library preparation kits and sequenced them at the FGCZ on HiSeq 2000 and HiSeq 2500 machines (short-insert libraries with a depth of 120x, mate-pair libraries with 26x). We quality filtered raw reads with Trimmomatic (Bolger et al., 2014) using default values for paired-end and single-end sequencing data. We assembled the genome with SOAPdenovo2 (Luo et al., 2012) using short-insert Illumina and 454 reads in the contig assembly step and 3 and 5 kb jumping libraries in the scaffolding step. We filled gaps in the final assembly using all 454 and Illumina data with Gap-closer (Luo et al., 2012). Our final assembly consisted of 13,000 scaffolds longer than 100 bp with an N50 value of ca. 100 kb and a total length of 340 Mbp. Genomic scaffolds can be found in Supplementary Data S1. Quality of the genome assembly was assessed by searching translated peptide sequences of the reconstructed gene set (see in section “Read Mapping and Quantification of Expression”) against hidden Markov models of Benchmarking Universal Single-Copy Orthologs (BUSCO) (Seppey et al., 2019). For this purpose, we used the Viridiplantae and Embryophyta datasets retrieved from OrthoDB v10.1 (Kriventseva et al., 2019). To put these results into context, publicly available gene coding sequences of *Marchantia polymorpha* (Bowman et al., 2017), *P. patens* (Lang et al., 2018), *Pleurozium schreberi* (Pederson et al., 2019), and *Sphagnum fallax* were retrieved from Phytozome v12 (Goodstein et al., 2012) and searched against the same BUSCO sets. Results of all completeness assessments are shown in Table 1. Our results show that the reconstructed gene set captures the complete sequence of 80.5% BUSCOs from the Embryophyta dataset and of 91.8% from the Viridiplantae dataset. These BUSCO values are close to the figures obtained for high-quality genome assemblies generated using long-read technologies (*M. polymorpha*, *P. patens*, and *S. fallax*) and clearly exceed the values of the *P. schreberi* genome, which was likewise assembled using only short-reads. Overall, our BUSCO analysis implies that the *F. hygrometrica* draft genome is of good quality.

**TABLE 1:** Assessment of completeness of the *F. hygrometrica* draft genome using Benchmarking Universal Single-Copy Orthologs (BUSCO).

	Complete			Fragmented	Missing
	Total	Single-copy	Duplicated		
**Embryophyta (*n* = 1614)**					
*F. hygrometrica*	1300(80.5%)	1101(68.2%)	199(12.3%)	43(2.7%)	271(16.8%)
*M. polymorpha*	1412(87.5%)	1385(85.8%)	27(1.7%)	20(1.2%)	182(11.3%)
*P. patens*	1423(88.1%)	1174(72.7%)	249(15.4%)	28(1.7%)	163(10.2%)
*P. schreberi*	736(45.6%)	540(33.5%)	196(12.1%)	278(17.2%)	600(37.2%)
*S. fallax*	1447(89.7%)	1257(77.9%)	190(11.8%)	22(1.4%)	145(8.9%)
**Viridiplantae (*n* = 425)**					
*F. hygrometrica*	390(91.8%)	336(79.1%)	54(12.7%)	7(1.6%)	28(6.6%)
*M. polymorpha*	411(96.7%)	409(96.2%)	2(0.5%)	3(0.7%)	11(2.6%)
*P. patens*	416(97.8%)	361(84.9%)	55(12.9%)	1(0.2%)	8(2.0%)
*P. schreberi*	227(53.4%)	160(37.6%)	67(15.8%)	107(25.2%)	91(21.4%)
*S. fallax*	417(98.1%)	375(88.2%)	42(9.9%)	2(0.5%)	6(1.4%)

*For comparison, BUSCO statistics for the *Marchantia polymorpha*, *Physcomitrium patens*, *Pleurozium schreberi*, and *Sphagnum fallax* genomes are shown. Data was retrieved from Phytozome v12 (Goodstein et al., 2012) and analyzed using the Viridiplantae and Embryophyta BUSCO datasets obtained from OrthoDB v10.1.*

### Read Mapping and Quantification of Expression

Mapping of the reads obtained by Illumina sequencing was performed following the HISAT2/StringTie pipeline (Pertea et al., 2016). First, adapter sequences were removed from the libraries and the reads were quality filtered using Trimmomatic v36 (Bolger et al., 2014) (ILLUMINACLIP:TruSeq3-SE.fa:2:30:10 SLIDINGWINDOW:4:5 LEADING:5 TRAILING:5 MINLEN:25). The reads were then mapped to the *P. patens* reference genome, version 3.1 (Lang et al., 2018), and the *F. hygrometrica* draft genome (Supplementary Data S1), respectively, using HISAT2 (Kim et al., 2015). Detailed statistics of the read mapping are available in Supplementary Table S1. To calculate transcript abundance for the *P. patens* data set, we used the version 3.1 gene annotation (Lang et al., 2018) in combination with quantification in StringTie. The transcriptome of *F. hygrometrica* was reconstructed using StringTie, including *de novo* assembly of transcripts and a subsequent quantification of transcript abundance (Pertea et al., 2015). To get a complete set of transcripts expressed during sporophyte development for each species, the assembled transcripts of all samples were merged using the –merge function of StringTie (Supplementary Data S1). The transcriptome reconstruction of *F. hygrometrica* yielded 25,904 unique loci. We discarded all gene models with a summarized read count over all samples lower than 10 to remove gene models with very few, potentially misaligned, reads. This filtering step reduced the gene set to 25,460 (*F. hygrometrica*) and 22,690 (*P. patens*) predicted gene models which show detectable expression in the analyzed samples.

### Preferential Expression in the Sporophyte Stage

Genes preferentially expressed in the sporophyte stage were identified using the R package DESeq2 version 1.16.1 (Love I, Huber and Anders, 2014). The sporophyte expression data comprised RNAseq raw count data from three replicates of four developmental stages each (for sporophyte developmental stage 1 from *P. patens* only two replicates were used). Expression data from the gametophyte phase included raw read counts from six (*F. hygrometrica*) and eight (*P. patens*) samples from different stages of gametophyte development. After defining sporophytic and gametophytic samples, a differential expression analysis was conducted using the unmodified DESeq2 algorithm. To obtain genes highly expressed in the sporophyte stage in contrast to gametophytic stages, significant results (*p*_adj_ ≤ 0.05) were filtered for gene models with a log_2_-fold change ≥2 (Supplementary Data S2, S3). We used conventional chi-square statistics to test for enrichment of genes in the set showing preferential sporophyte expression.

### Identification of Homologs and Orthologs

Homologous genes (including orthologs and paralogs) were identified by the BLASTp algorithm (Altschul et al., 1990) and the software Orthofinder2 (Emms and Kelly, 2018). We used this approach to identify *F. hygrometrica* and *P. patens* gene models that are species-specific (i.e., that have no detectable homolog in the alternate species’ proteome). Besides Orthofinder, we used BLASTp to detect distant homologs that might be missed by the more stringent approach of Orthofinder. The BLASTp search included reference peptide sequences for each gene model in the *P. patens* and *F. hygrometrica* data sets. Hits were filtered for alignments with excess similarity by applying an *E*-value threshold of ≤10^–6^. To remove alignments that cover only small conserved domains within the query we applied a second filtering step for alignments with ≥80% query coverage and ≥35% sequence similarity between the query and the corresponding hit. Both filtering methods are reported to reliably detect homologous sequences even between distantly related species (Rost, 1999; Pearson, 2013). We also ran Orthofinder2 v2.3.3 (Emms and Kelly, 2018) to identify orthogroups (comprising both orthologs and paralogs) between *P. patens* and *F. hygrometrica* using default parameters (Supplementary Data S4). We included the following proteomes to generate orthogroups: *Citrus clementina, M. polymorpha, Theobroma cacao, Vitis vinifera, Prunus persica, Cucumis sativus, Amborella trichopoda, Physcomitrium patens, F. hygrometrica, Selaginella moellendorffii, Zea mays, Oryza sativa v7_JGI, Brassica oleracea, Arabidopsis thaliana, P. trichocarpa, Medicago truncatula*, and *Daucus carota*. All proteomes, except the one for *F. hygrometrica*, were retrieved from Phytozome v12 (Goodstein et al., 2012). We also let Orthofinder2 v2.3.3 automatically calculate gene trees for each orthogroup and identify one-to-one orthologs of *P. patens* and *F. hygrometrica* using phylogenetic information. We used one-to-one orthologs between *P. patens* and *F. hygrometrica* to compare gene expression in the two species.

### PCA

**FIGURE 3:**
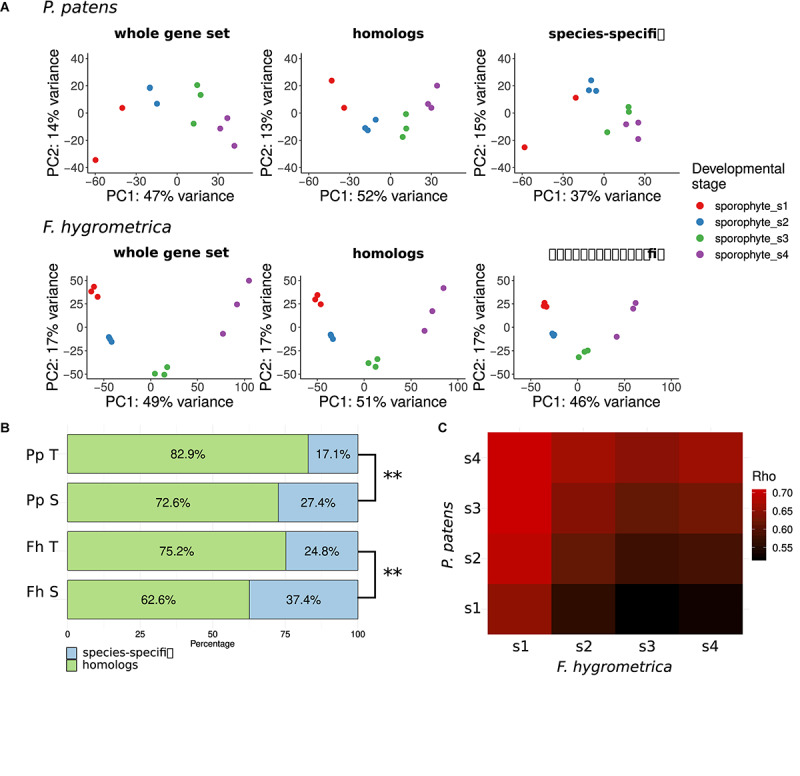
Gene expression variation throughout the sporophyte development of *Funaria hygrometrica* and *Physcomitrium patens*. **(A)** Principal component analysis (PCA) of gene expression variation during sporophyte development in *P. patens* and *F. hygrometrica*. PCA was carried out by using gene expression data for all expressed genes (“whole gene set”), for homologous genes (“homologs”), and for species-specific genes (“species-specific”). **(B)** Proportion of homologous and species-specific genes in the transcriptome data sets of *F. hygrometrica* (Fh) and *P. patens* (Pp). For each species the total set of transcripts detected in the gametophyte and sporophyte phases (Pp T, Fh T) and the set of transcripts that is specifically expressed in the sporophyte phase (Pp S, Fh S; log_2_ fold change ≥ 2, *q* ≤ 0.05) is shown. ^∗∗^ marks significant dependence of distribution between homologs and species-specific genes on the considered subset according to Chi-squared test (*p* ≤ 0.01). **(C)** Correlation coefficients (Spearman’s Rho) of gene expression between developmental stages 1 to 4 of *P. patens* and *F. hygrometrica* sporophytes. Only one-to-one orthologs for which expression was observed in both samples were used to calculate the corresponding rank correlation. Spearman’s Rho was calculated using normalized and log-transformed raw count values.

To gain insights into the overall change of gene expression associated with sporophyte development in *F. hygrometrica* and *P. patens*, we used principal component (PCA) analyses (Hotelling, 1933) on normalized and standardized RNA-seq expression data (Figure 3A). Because we assumed that divergent sporophyte morphologies may be partially associated with gene gain/loss we generated PCAs separately using each species’ full gene set (including both homologs and species-specific genes), the gene set with homologs in the alternate species (including orthologs and paralogs) and the species-specific gene set (non-homologs, for details see section “Identification of Homologs and Orthologs”). We argue here that the amplitude of a gene’s expression variation (expression dynamics) throughout sporophyte developmental stages likely correlates with its functional relevance. Following this reasoning, we expected more pronounced differentiation across sporophyte developmental stages in gene expression using the species-specific gene set if gene gain/loss is more important in contributing to the divergent sporophyte morphologies than genes that are part of the shared gene set (homologous genes). Alternatively, expression variation across sporophyte developmental stages may be more pronounced using the shared gene set if sporophyte development is rather driven by gene expression changes in homologous genes and not the expression dynamics of species-specific genes.

To further investigate which sporophyte developmental stages showed similar or rather divergent gene expression patterns in the two species we calculated an expression divergence matrix between developmental stages, using one-to-one orthologs that showed expression in both species in the corresponding stage. The expression divergence matrix was built by calculating pairwise Spearman’s rank correlation coefficients between expression profiles of developmental stages of *F. hygrometrica* and *P. patens*. Results were plotted using the R package ggplot2 v. 3.2.1 (Wickham, 2009).

### Functional Annotation

We retrieved functional annotation of *P. patens* gene models from Phytozome v11 (Goodstein et al., 2012). We translated and annotated transcript sequences of *F. hygrometrica* by running tBLASTn searches (*E*-value threshold 10^–6^) against the plant proteomes available in PLAZA v2.0 (Proost et al., 2009). GO annotation for each transcript was obtained by transferring the GO annotation of the respective best hit protein to the *F. hygrometrica* transcript.

Key positions in gene regulatory networks are often occupied by transcription factors, which trigger expression of an array of downstream target genes resulting in a regulatory cascade setting off developmental transitions (Davidson and Erwin, 2006; Erwin and Davidson, 2009). Identification of gene models that encode transcription factors was done based on conserved DNA binding motifs using the PlantTFDB 4.0 resource (Jin et al., 2017). The algorithm reported 1,061 (*P. patens*) and 849 (*F. hygrometrica*) transcription factor encoding genes, which are expressed in at least one sample.

### Gene Ontology Term Enrichment

**FIGURE 4:**
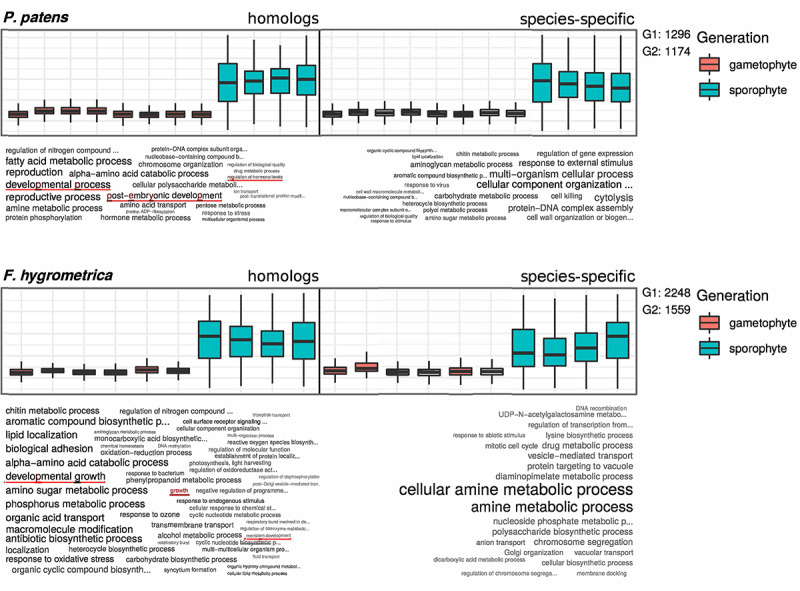
GO term enrichment analysis of preferentially sporophyte expressed genes in *Physcomitrium patens* and *Funaria hygrometrica.* Upper panels show standardized expression values of the respective subset of genes. Homologous genes are shown on the left side and species-specific genes on the right side of the plots. Lower panels show a visualization of enriched GO terms in the respective subsets, word size is correlated with the significance value obtained from the enrichment analysis. G1 and G2 denote the number of genes in the subsets of homologous (G1) and species-specific (G2) genes, used in the enrichment analysis. GO terms potentially connected to differential sporophyte development in the two species are underlined (see red lines).

To functionally characterize genes preferentially expressed in the sporophyte phase of *F. hygrometrica* and *P. patens*, we used the R package TopGO, version 2.38.1 (Kim, 2019). For the enrichment analyses we only considered GO-terms of the class “Biological Process.” After defining all gene models with detectable expression in the RNA-seq data set of sporophyte and gametophyte tissue as the gene universe, and identifying all genes which are preferentially expressed in the sporophyte phase of either species as the subset of interesting genes, enriched GO-terms were computed using the Parent-Child Algorithm (Grossmann et al., 2007) and their significance tested with Fisher’s exact test, both implemented in the TopGO package. Lists of enriched GO-terms (Supplementary Data S5–S8) were reduced using the REVIGO online resource (Supek et al., 2011) to remove redundant terms. Results of the enrichment analysis (Figure 4) were visualized using the R package GOsummaries version 2.22.0 (Kolde and Vilo, 2015).

### Clustering by Gene Expression Profiles

The main goal of this study is to identify the genes and gene networks that may have contributed to the divergent development of the sporophyte phase in *P. patens* and *F. hygrometrica*. Since development is a dynamic, time dependent process we sought to use a method that would allow us to statistically distinguish one-to-one orthologous gene sets showing differential expression dynamics throughout sporophyte development between *P. patens* and *F. hygrometrica*. To compare expression dynamics of one-to-one orthologous genes in the two species during sporophyte development, we used a method enabling classification of genes by their expression pattern and mapping gene expression profiles of one species against clusters of the other species. To achieve this, we carried out fuzzy c-means clustering with the R package mfuzz (Kumar and Futschik, 2007). We first clustered the *F. hygrometrica* gene expression data and then statistically assigned genes of *P. patens* to these clusters according to their expression dynamics. Because this assignment relies on the alignment of putatively functionally conserved genes, the analysis is restricted to one-to-one orthologs as detected by the Orthofinder algorithm (see section “Materials and Methods”). To obtain the optimal cluster number, we computed the minimum centroid distance for a range between four and 40 clusters using the implemented Dmin function. Centroid distances decreased rapidly up to a cluster number of 12. For higher cluster numbers the distance curve flattened out and changes in centroid distances were less severe. Therefore, we decided to use 12 clusters for the fuzzy c-means clustering as a compromise between describing the expression dynamics reasonably well without obtaining too many redundant clusters. The optimal fuzzifier was computed according to the method described by Schwämmle and Jensen (2010), which is implemented in the mfuzz package (mestimate function). Assignments of *F. hygrometrica* and *P. patens* one-to-one orthologs to the clusters can be found in Supplementary Data S9, S10.

## Results

To identify genes potentially underlying the difference in sporophyte development between *F. hygrometrica* and *P. patens*, we measured gene expression across four distinct stages of sporophyte development and several stages of gametophyte development in both species using RNA-sequencing (Figure 2). We assumed that most genes expressed or preferentially expressed in the sporophyte phase are important for proper sporophyte development. These data form the basis of our analyses described below.

### Proportion of Preferentially Sporophyte Expressed Genes Is Slightly Higher in the Species With Greater Sporophyte Complexity

First, we performed a quantitative and qualitative assessment of the gene set expressed during sporophyte development in both species. After applying a relaxed expression threshold (see section “Materials and Methods”), 22,690 *P. patens* gene models were considered to be expressed in at least one of the sporophyte developmental stages, which is about 64% of the 35,307 currently annotated gene models (Lang et al., 2018). Similarly, of the total 25,904 gene models assembled using RNA-seq data of *F. hygrometrica* (see section “Materials and Methods”), 25,460 (98%) showed expression during sporophyte development. Of the gene set expressed during sporophyte and gametophyte development, 2,807 (12.4%) (*P. patens*) and 3,807 (15%) (*F. hygrometrica*) gene models were strongly upregulated in the sporophyte versus the gametophyte phase (log_2_ fold change ≥2, false discovery rate ≤0.05). This suggests that the proportion of preferentially sporophyte expressed genes is slightly higher in the species with a more complex sporophyte morphology, but such genes represent only a relatively small proportion of the total gene set in both species.

### Sporophytic Transcriptomes of *P. patens* and *F. hygrometrica* Are Dominated by Homologs but the Contribution of Species-Specific Genes Is Not Negligible

We then asked whether transcripts expressed during sporophyte development mainly represent genes homologous (including orthologs and paralogs, see identification of homologs in section “Identification of Homologs and Orthologs”) between the two species or, instead, species-specific genes. The Orthofinder analysis revealed that 19,667 and 19,267 genes of *P. patens* and *F. hygrometrica* fall into orthogroups containing sequences from both species. That is 55% (19,667 out of 35,307) and 74% (19,267 out of 25,904) of the *P. patens* and *F. hygrometrica* full gene set had homologs (orthologs and paralogs) in the alternate species’ proteome, respectively. Out of these homologs, 12,741 gene models were identified as one-to-one orthologs. When expressed as a proportion of genes transcribed throughout sporophyte development in *P. patens* and *F. hygrometrica*, 85.3% (19,364 of 22,690 gene models) and 75.6% (19,245 of 25,460 gene models), respectively, had homologs in the alternate species’ genome. These numbers did not change considerably when using the more relaxed BLASTp approach (see section “Identification of Homologs and Orthologs”) to include more distant homologs. To ensure that genes without homologs in either one of the species are species-specific and not missed by the annotation process, we searched protein sequences of putatively non-homologous genes against the corresponding genome sequence, using the tBLASTn algorithm (Camacho et al., 2009) with a threshold of ≥80% query coverage and ≥35% sequence similarity between the query and the corresponding hit. We identified an additional 2,003 gene models of *P. patens* to have putative homologs in the *F. hygrometrica* genome, which was expected due to the fact that the gene models for *F. hygrometrica* were assembled using RNAseq data and genes, which are not expressed in the sampled stages will not be detected by this method. Similarly, 980 additional gene models in the *F. hygrometrica* gene set have homologs in the *P. patens* genome. Combined with the expression data, 81% and 78% of the sporophytic transcriptome of *P. patens* and *F. hygrometrica*, respectively, thus have homologs in the alternate species’ genome. In summary, the two species express a large proportion of homologous (about 80%) genes during the development of their sporophyte. Nevertheless, about 20% of their genes expressed during sporophyte development appear species-specific, that is with no clear homologs in the other species’ genome.

### *F. hygrometrica* Shows More Expression Variation During Sporophyte Development Than *P. patens*

Sporophyte development stages showed distinct expression patterns in *F. hygrometrica* in the shared (orthologs and paralogs: homologs) and species-specific gene sets (Figure 3A). By contrast, differentiation in gene expression among the four sporophyte developmental stages in *P. patens* was strongly dependent on the gene set used. They were well-differentiated when we used all genes or only the set of homologs, whereas only the early and the late two developmental stages were well-distinguishable when comparing expression of species-specific genes (Figure 3A).

### Species-Specific Genes Are Overrepresented Among Preferentially Sporophyte-Expressed Genes

Next, we tested how species-specificity or shared nature of genes is correlated with their putative function in sporophyte development. We assumed that preferential expression of genes in the sporophyte stage (compared to the gametophyte) can be used as a proxy for their functional role in sporophyte development. During gametophyte and sporophyte development 24,031 and 25,818 gene models were expressed in *P. patens* and *F. hygrometrica* of which 82.9% and 75.2% fell into orthogroups containing genes from both species, respectively. When using genes preferentially expressed in the sporophyte phase (log_2_ fold change ≥2, false discovery rate ≤0.05), the proportion of genes coming from orthogroups shared by the two species decreased: only 72.6% (*P. patens*, 1,792 gene models) and 62.6% (*F. hygrometrica*, 2,385 gene models) of preferentially sporophyte expressed genes were homologous between the two species (Figure 3B). This difference in the proportion of homologous genes between preferentially sporophyte expressed and all expressed genes was highly significant in both species according to a chi-squared test (*P. patens*: χ^2^_df__=__1_ = 206.16, *p* < 2.2 × 10^–16^_;_ *F. hygrometrica*: χ^2^_df__=__1_ = 384.73, *p* < 2.2 × 10^–16^). Altogether, this implies that species-specific genes are significantly overrepresented while homologous genes are underrepresented in the gene set preferentially expressed in the sporophyte.

Comparing gene expression at specific developmental stages between the two species (Figure 3C) using one-to-one orthologs reveals that all sporophytic stages of *P. patens* were most similar to the earliest sporophyte developmental stage of *F. hygrometrica* (stage number 1, see Figure 3C). Furthermore, overall expression similarity increased between *P. patens* and any of the *F. hygrometrica* developmental stages along a developmental chronology with the expression in the first stage of *P. patens* being most dissimilar. This suggests that the major differences in gene expression of orthologs can be found during early sporophyte development, a stage where precursors of various tissues that will form the mature sporophyte are likely established (Schwartz, 1997).

### Functional Analysis of Genes Suggests a Contribution of Homologs in Establishing Contrasting Sporophyte Phenotypes

Comparing GO-terms of species-specific and homologous genes preferentially expressed in the sporophyte stage of both species (see section “Materials and Methods” for details) revealed that 103 (homologs) and 32 (species-specific) GO terms in *F. hygrometrica* and 55 (homologs) and 50 (species-specific) GO terms in *P. patens* were significantly enriched in the corresponding subset (Fisher’s exact test, *p* ≤ 0.05, Figure 4). The majority of enriched GO-terms is related to various metabolic processes in both species. Nevertheless, some significantly enriched GO terms are potentially linked to the establishment of sporophyte morphology, which among others includes terms such as “regulation of growth”, “developmental transitions”, and “structure morphogenesis” (Figure 4). Importantly, only the homologous gene set was enriched for GO terms potentially linked to the morphology and development of the sporophyte phase in both species investigated (Figure 4). By contrast, the species-specific subset of preferentially sporophyte expressed genes were primarily enriched for general metabolic processes. In particular, homologous genes preferentially expressed in the sporophyte of *P. patens* were enriched for terms “developmental process,” “post-embryonic development,” and “regulation of hormone levels.” Similarly, homologous genes preferentially expressed in the sporophyte in *F. hygrometrica* were enriched for GO-terms “developmental growth,” “growth,” and “meristem development” that are possibly linked to the establishment of the complex sporophyte morphology. By contrast, species-specific genes preferentially expressed in the sporophyte phase in both species were primarily enriched for GO terms related to general metabolic processes and not to differential development of sporophytic structures. Furthermore, while about 50% of the preferentially sporophyte expressed homologs in both species were associated with at least one GO-term, only 12% (*F. hygrometrica*) and 22% (*P. patens*) of the species-specific genes could be functionally characterized by one or more GO-terms, and consequently the contrasting pattern of homologous and species-specific genes may result from their sparse functional annotation.

### Several Orthologous Developmental Regulators Show Heterochronic Expression in *P. patens* and *F. hygrometrica*

**FIGURE 5:**
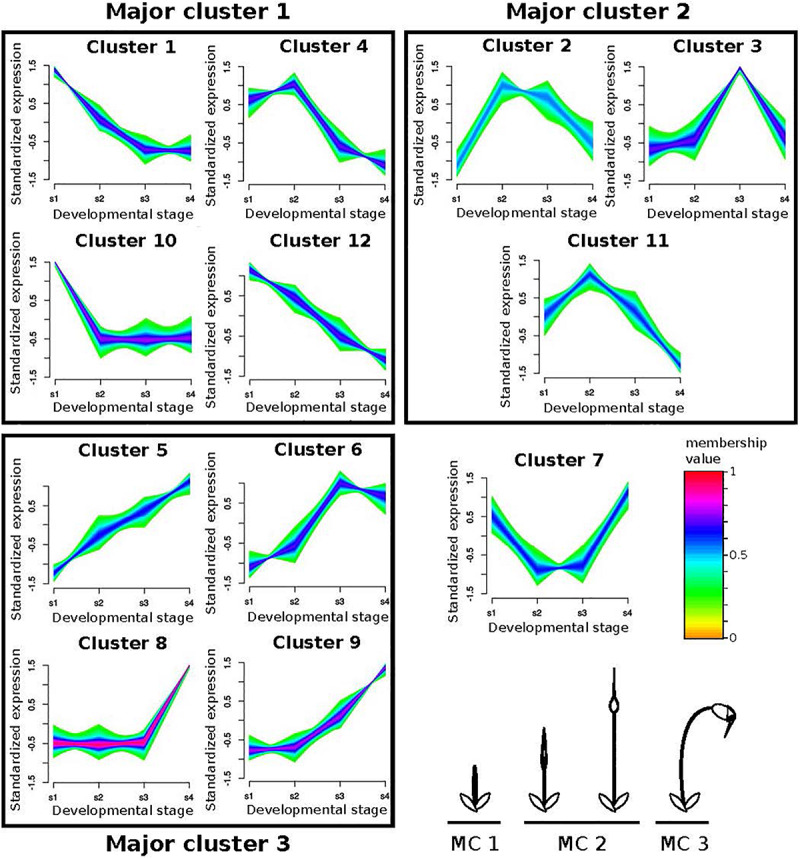
Fuzzy clustering of gene expression data from 4 stages of sporophyte development of *Funaria hygrometrica* and *Physcomitrium patens*. We identified 12 clusters with distinct expression profiles. For each cluster, relative expression changes of the assigned genes are shown together with their color-coded membership values, indicating how strong their expression pattern correlates with the cluster core. The clusters are further condensed to Major clusters (MC), depending on whether expression peaks occurred during early (MC 1), intermediate (MC 2), or late (MC 3) sporophyte development (bottom right). Cluster 7 could not be assigned to one of the major clusters. s1 – s4: sporophyte developmental stages 1 to 4.

We used fuzzy c-mean clustering to identify groups of one-to-one orthologous genes experiencing differential expression dynamics throughout sporophyte development between *P. patens* and *F. hygrometrica*. We first clustered the *F. hygrometrica* gene expression data and then statistically assigned genes of *P. patens* to these clusters according to their expression dynamics. Clustering genes according to their expression profile over sporophyte development in *F. hygrometrica* resulted in 12 clusters with distinct expression patterns (referred to as minor clusters). We assigned those clusters to early, mid, and late sporophyte development (referred to as major clusters), based on the expression peak of each cluster (Figure 5). Following filtering (see section “Materials and Methods”), the data set contained 3,976 one-to-one orthologs assigned to one of the minor clusters. Of these, 1,807 (45.4%) were assigned to the same major cluster in both species, and 2,169 (54.6%) to different major clusters (Supplementary Data S9).

Looking at only transcription factor encoding genes did not influence our conclusion. Half of the 230 transcription factor encoding one-to-one orthologs present in the analyzed data set (i.e., 114, 51.8%) shifted between major clusters in *P. patens* and *F. hygrometrica* whereas the other half (i.e., 106, 48.2%) stayed in the same major cluster. These proportions were not significantly different from those when considering all expressed genes. Among the differentially expressed transcription factor encoding genes, many are members of gene families frequently associated with developmental control and growth in plants, including AP2/ERF, ARF, GRAS, MADS-box, TALE and WOX (see Supplementary Data S10 for a complete table).

## Discussion

Morphological novelties may be associated with gene birth/death (*de novo* evolution of genes or loss of genes) or can alternatively be achieved by existing genes acquiring a new function (Davidson, 2010; Pires and Dolan, 2012; Chen et al., 2013; Schlötterer, 2015; Li et al., 2016; Das Gupta and Tsiantis, 2018; Bowman et al., 2019; Zhang et al., 2019). The relative contribution of these two major processes to the evolution of morphological novelties is still poorly known. Here, we compared the transcriptomes of two moss species, *P. patens* and *F. hygrometrica*, to investigate the relative contribution of gene gain/loss and shifts in expression dynamics of orthologous genes to the contrasting morphologies of their sporophyte phase. We found that the divergent sporophyte morphologies are primarily achieved by the heterochronic expression of a conserved set of genes, and while species-specific genes are likely to be also important, their contribution remains to be clarified. Our study contributes to the growing set of observations suggesting that shifting temporal dynamics of conserved genes represents a frequent mechanism through which radically different morphologies can be achieved (Sakamoto et al., 2009; Pires and Dolan, 2012; Lemmon et al., 2016; Olson and Nedelcu, 2016; Pham et al., 2017; Fisher et al., 2019). Moreover, it provides critical information on the molecular processes contributing to the diversification of moss sporophytes, a central topic of bryophyte taxonomy and evolutionary research. In the following paragraphs we discuss the implications of our findings for the molecular mechanisms underlying divergent sporophyte morphologies in *P. patens* and *F. hygrometrica* and for the evolution of morphological novelties in general.

### Proportion of Preferentially Sporophyte-Expressed Genes Is Small

Developmental processes regulating the morphological properties of organisms are known to be composed of an interconnected set of genes organized in so-called regulatory networks (Davidson and Erwin, 2006; Davidson, 2010; Das Gupta and Tsiantis, 2018). Regulatory networks are modular, which makes them highly evolvable through the replacement of conserved modules and the rewiring of regulatory connections (Rosin and Kramer, 2009; Glassford et al., 2015; Halfon, 2017; Verd et al., 2019). Although many genes are expressed during sporophyte development in both species, only about 12–15% indeed exhibit preferential sporophytic expression and are thus likely components of the genetic network underlying the development of the sporophyte versus the gametophyte. Consequently, shifts from the gametophyte to the sporophyte program would only require changes in expression of a relatively small set of major genes. This is in line with multiple studies reporting how shifts in temporal or local expression of a small set of genes can lead to radical change in morphology (Frankel et al., 2011; Ichihashi et al., 2014; Sicard et al., 2014; Di Ruocco et al., 2018; Whitewoods et al., 2020). Whether this assertion may also apply to the molecular mechanisms underlying contrasting sporophytic architectures is discussed in the paragraphs below.

### Evidence for Control of Divergent Sporophyte Development by Conserved Orthologs Is Mounting, but Contribution of Species-Specific Genes Remains Unclear

Novel morphological structures can be acquired by the evolution of new genes or the differential usage of already existing gene sets, but the significance of either mechanism in shaping innovation or shifts in morphology remains ambiguous, and likely spans a broad range across lineages (Davidson, 2010; Pires and Dolan, 2012; Chen et al., 2013; Schlötterer, 2015; Li et al., 2016; Das Gupta and Tsiantis, 2018; Bowman et al., 2019; Zhang et al., 2019). Although the body plan of the moss sporophyte is fundamentally simple (Goffinet and Buck, 2013), the complexity of its mature architecture varies considerably (Vitt, 1981), and homoplastic reduction seems to be common across mosses (Schwartz, 1994; Shaw et al., 2000).

Within the Funariaceae the architecture of the sporophyte varies considerably, with *P. patens* and *F. hygrometrica* representing the two extremes of the spectrum (Liu et al., 2012; Medina et al., 2018, 2019). Their large-scale morphological difference lies in the length of the seta, the (a)symmetry of the spore bearing capsule and the presence/absence of appendages controlling spore dispersal (Fife, 1982). Sporophytes of *F. hygrometrica* consist of a 2–4.5 cm long stalk (seta) bearing an asymmetric capsule with subapical differentiated cells (annulus) enabling the release of a lid (operculum), and the then exposed mouth is lined by small hygroscopic teeth (peristome). By contrast, the sporophyte of *P. patens* is composed of an extremely short stalk, ending in a spherical capsule whose epidermal wall disintegrates when spores are mature (Figure 2).

Our study provides the first hints on the molecular mechanisms potentially underlying contrasting sporophyte architectures in *P. patens* and *F. hygrometrica*. We found that about half of the one-to-one orthologs between *P. patens* and *F. hygrometrica* show divergent expression dynamics during sporophyte development of the two species as demonstrated by our clustering analysis. Furthermore, of the transcription factor encoding orthologs that show heterochronic expression, many are members of gene families frequently associated with developmental control and growth in plants, including AP2/ERF, ARF, GRAS, MADS-box, TALE and WOX (see Supplementary Data S10 for a complete table). Finally, our gene ontology analysis and previous functional studies on *P. patens* also provided evidence that some of these genes are likely to be key players in contributing to the divergent sporophyte phenotypes. Altogether, our data supports the notion that drastic macromorphological differences in sporophyte morphology (seta length and opening mechanisms of the capsule) are likely achieved by the heterochronic expression of orthologous transcription factors.

Besides the overwhelming support for the contribution of orthologous transcription factors to the two contrasting sporophyte architectures, our analysis provides strong evidence that species-specific genes are also fundamental. We found that a large proportion of all (roughly 20%) and preferentially sporophyte expressed genes (between 30 and 40%) had no orthologs/homologs in the alternate species’ genome and thus were truly species-specific. The proportion of species-specific genes between *P. patens* and *F. hygrometrica* appears to be high but not exceptional when considering their estimated divergence time of 60 million years (Medina et al., 2018). Comparable values can also be observed between closely related species pairs in angiosperms. For instance, *Arabidopsis thaliana* and *Arabidopsis lyrata* diverged 10 million years ago and roughly 16–18% of their gene sets are species-specific, respectively (Hu et al., 2011). Similar proportion of species-specific genes were also observed between the sister species *Zostera marina* and *Zostera muelleri*, which diverged from one another roughly 14 million years ago (Lee et al., 2016; Olsen et al., 2016). We further found that genes upregulated in the sporophyte phase were significantly enriched for species-specific genes suggesting their preferential recruitment for sporophytic functions. Unfortunately, species-specific genes are poorly annotated and thus their specific contribution in establishing divergent sporophyte architectures remains unclear and awaits clarification. In the next two paragraphs, we will focus on some major morphological features and their suggested molecular basis supported by our analyses.

### Molecular Basis of Differential Seta Length in *P. patens* and *F. hygrometrica*

The seta develops through the activity of an intercalary meristem located immediately below the presumptive tissue of the capsule (Garner and Paolillo, 1973; French and Paolillo, 1976; Sano et al., 2005; Figure 2). The intercalary meristem is basipetal, such that cells are added below it and elongating, contributing to the growth of the seta. Thus, the length of the seta is likely correlated with the duration of activity of the intercalary meristem during sporophyte development. Given that the sporophyte of *P. patens* completes its development faster than *F. hygrometrica* (French and Paolillo, 1975; Sano et al., 2005; Coudert et al., 2019) as its meristem soon ceases to add new cells, it is therefore likely that expression of the underlying regulatory networks is shut down sooner or shortened compared to *F. hygrometrica*. That is, heterochronic expression of the meristem activity regulatory network can contribute to the seta length difference.

The class I KNOX gene *PpMKN2* promotes sporophyte axis extension by activating cytokinin biosynthesis pathways in *P. patens* (French and Paolillo, 1975; Coudert et al., 2019). In this species, *PpMKN2* is mainly expressed in the first sporophyte stage with expression quickly decreasing in more advanced stages of sporophyte development (Figure 5, cluster 10). By contrast, Fh_22445, the *F. hygrometrica* ortholog of *PpMKN2*, is characterized by a slower decline of expression reaching its minimum in the third and fourth phases of sporophyte development (Figure 5, cluster 1). The apparent correlation between difference in gene expression and degrees of seta length in the two species may suggest that the prolonged expression of the class I KNOX ortholog in *F. hygrometrica* accounts for the elongated seta phenotype.

A WUSCHEL-related homeobox13-like (WOX13-like) gene in *P. patens* (Pp3c15_20000) and its ortholog Fh_3609 also display differential gene expression dynamics in the two species. Fh_3609 is equally highly expressed during the first two developmental stages, but only weakly in other stages (Figure 5, cluster 4), whereas Pp3c15_20000 reaches an expression maximum only during the second stage of sporophyte development in *P. patens* (Figure 5, cluster 11). WOX13-like genes are important factors in initiating cell growth during zygote and stem cell formation by upregulating genes controlling cell wall loosening, including expansins (Sakakibara et al., 2014). This coincides with our finding that growth related genes are enriched in the set of preferentially sporophyte expressed genes in *F. hygrometrica*, among them three expansin homologs (Fh_21099, Fh_21985, Fh_4588). Furthermore, all of these expansins were assigned to the same major cluster as the *WOX13-like* gene Fh_3609 and two of them to the very same minor cluster. The three expansin homologs also have co-orthologous sequences in *P. patens* (Pp3c8_15200, Pp3c20_5780, Pp3c21_22300), but only Pp3c8_15200 is co-expressed with the *WOX13-like* gene Pp3c15_20000, whereas the other two show opposite expression patterns reaching their maximum expression in developmental stage 4 (Figure 5, cluster 5). Taken together, these results suggest that additional genes aiding in cell expansion and growth were recruited to the regulatory module initiated by expression of the *WOX13-like* gene Fh_3609 in *F. hygrometrica*, presumably to facilitate the rapid growth of the seta during early sporophyte development. Alternatively, regulatory connections between the *WOX13-like* gene of *P. patens* and some expansin homologs could have been lost in *P. patens*, contributing to the reduced growth of the seta.

### Regulators of Abscission and Dehiscence Pathways Could Fulfill a Similar Function in Peristome and Annulus Development in *F. hygrometrica*

In addition to their highly contrasted seta length, the sporophytes of *F. hygrometrica* and *P. patens* differ in the presence/absence of specialized structures aiding in spore release and dispersal (Figure 2). The sporangium of *F. hygrometrica* opens via the loss of a lid (operculum), enabled by the differentiation of an annulus, a subapical ring of cells forming a predetermined breaking point between capsule body and operculum (Garner and Paolillo, 1973). The release of the operculum exposes the capsule mouth, which is lined by two rows of hygroscopic appendages, the peristome teeth, whose movement over the mouth at least partially controls the dispersal of spores. The development of both structures, the annulus and peristome teeth, requires the establishment of tissue layers, spatially and temporally tightly regulated cell death and cell wall break down during development of the sporophyte (Budke et al., 2007; Goffinet et al., 2009; Goffinet and Buck, 2013), a process reminiscent of abscission and dehiscence found in a wide variety of plant species.

Control of abscission and dehiscence on a genetic level are well studied in vascular plants (Lenser and Theißen, 2013; Kim et al., 2019), but regulatory pathways remain unexplored in bryophytes. Central regulators of abscission zone (AZ) formation in *Arabidopsis thaliana* are the redundantly acting transcription factors *BLADE-ON-PETIOLE1* (*BOP1*) and *BOP2* (McKim et al., 2008; Wu et al., 2012; Khan et al., 2014), for which co-orthologs are present both in *F. hygrometrica* (Fh_25624, Fh_20164, Fh_1793) and *P. patens* (Pp3c17_8330, Pp3c1_35410, Pp3c14_11190). In *P. patens* the BOP co-orthologs show expression throughout sporophyte development with peaks in stages 1, 3 and 4, respectively. By contrast, expression of the *F. hygrometrica* BOP co-orthologs is more focused to intermediate developmental stages with Fh_25624 and Fh_1793 displaying peak expression in stage 2 and Fh_20164 being most strongly expressed in stage 3. The differentiation of capsule tissues begins after the seta has reached its maximum length (Garner and Paolillo, 1973), which happens around developmental stage 3. The shift of *F. hygrometrica* BOP ortholog expression to intermediate developmental stages when the capsule is not yet differentiated (stage 2) and differentiation begins (stage 3) could point to a potential role of these genes in establishing the boundary tissues that will later allow detachment of the operculum and formation of peristome teeth. However, BOP transcription factors are also involved in various other developmental and physiological processes in plants (Khan et al., 2014) and no apparent phenotypes in the sporophyte were reported in mutant analyses of PpBOP genes (Hata et al., 2019). Therefore, functional studies of BOP co-orthologs in *F. hygrometrica* are necessary to determine if these genes play a role in capsule tissue patterning that has been lost in funarioid and potentially also other species with a reduced sporophyte phenotype.

We also identified a pair of MIKC^C^-type MADS-box gene orthologs in *F. hygrometrica* (Fh_2640) and *P. patens* (Pp3c14_14900), which differ in their expression profile throughout sporophyte development. Fh_2640 shows an expression peak during late stages of sporophyte development (stage 3 and 4) whereas Pp3c14_14900 is primarily expressed during the elongation phase in stages 2 and 3. This shift in expression likely represents a change in the regulatory context of the orthologs, but whether this change is also accompanied by a functional diversification is currently unclear. MIKC^C^-type MADS-box genes are both expressed in the gametophyte and sporophyte phases in *P. patens* and are redundant negative regulators of cell division and growth in the gametophyte as well as sperm formation (Koshimizu et al., 2018). Their function in sporophyte development of mosses is poorly understood and debated. Koshimizu et al. (2018) could not identify a phenotypic effect in knockout lines on sporophyte development in *P. patens* although all six MIKC^C^ MADS-box genes were strongly expressed in the sporophyte phase. By contrast, other studies reported well-recognizable sporophyte phenotypes when down regulating some MIKC^C^ genes in *P. patens* (Tanabe et al., 2003; Singer et al., 2007). We speculate that the MIKC^C^-type MADS-box genes may also be important regulators of sporophyte development, potentially as negative regulators of cell division, growth and tissue patterning. The temporal expression shift of the *F. hygrometrica* ortholog to later stages of development may play a significant yet currently unknown role in the evolution of the more elaborate sporophyte morphology. This assumption is supported by the observations that down regulation of MIKC^C^-type genes in *P. patens* led to abnormal swelling of the capsule (Singer et al., 2007) and that fluorescent reporter lines in *P. patens* showed that MIKC^C^-type MADS-box genes have specific and complementary expression patterns throughout the development of the sporophyte. Altogether, it is possible that temporal expression shifts of MIKC^C^-type MADS-box genes have contributed to the morphological diversification of sporophytes in Funarioid mosses, which parallels the observation that MIKC^C^-type genes are key regulators in various aspects of sporophyte development including the diversification of flowers in angiosperms (Gramzow and Theissen, 2010). Nevertheless, sporophytic function of the MIKC^C^-type genes in *P. patens* and *F. hygrometrica* must be determined in future studies on sporophyte development.

### Implications for the Evolution of Sporophytic Characters in Mosses

The sporophyte is responsible for the production of spores, which will initiate the development of a new free-living gametophytic generation. Transformations of the sporophyte during the diversification of mosses affect a broad spectrum of traits, such as spore numbers, asymmetry of the capsule, mode of dehiscence, length of the seta, presence of stomata, responses of the capsule wall to dehydration, or architecture of the peristome controlling spore release (Crum, 2001). All these modifications may have a direct impact on fitness and are thus likely under natural selection (Vitt, 1981; Rose et al., 2016). Although some mosses have atypically amplified sporophytic traits [e.g., entomophilous Splachnaceae (Marino et al., 2009), or *Buxbaumia* (Crum, 2001)], and the macroevolutionary trend is one of increased complexity, repeated reductions through loss of complexity is rampant across the diversification of mosses, leading to capsules being immersed among vegetative leaves, lacking a complete peristome, stomata, or an operculum. Reduced sporophytic architecture characterizes many lineages distributed along a decreasing humidity gradient, and is common among epiphytic mosses, and particularly among xerophytic or short-lived annual mosses (Vitt, 1981; Rose et al., 2016). Finally, given the phylogenetic distribution of taxa with reduced morphology among congeners with more complex architectures, such as the three species traditionally referred to as *Physcomitrella* (highly reduced sporophytes) scattered among species of *Physcomitrium* (with more elaborate sporophytes; Medina et al., 2019), reduction may result from repeated breakdown or shut-down of complex traits, and thus convergence may be readily achieved. Reduced sporophyte complexity of *P. patens* may be easily achieved by loss or reduced expression of particular *F. hygrometrica* genes involved in sporophyte development. This is in line with previous observations that temporal and/or spatial shifts in gene expression have significantly contributed to morphological diversification both in animal and plant systems (Frankel et al., 2011; Ichihashi et al., 2014; Rast-Somssich et al., 2015; Das Gupta and Tsiantis, 2018).

Our comparative study of transcriptomic profiles of developing sporophytes in *Physcomitrium patens* and *Funaria hygrometrica* provides the very first insights into the potential genetic tools shaping sporophyte morphologies. Our observations reveal that species-specific genes are preferentially expressed during sporophyte development in both species. While the actual function of these genes is poorly known, they may be involved in meiotic processes, physiological processes such as those related to endohydric, or metabolic pathways such as for components of the cuticle (Koch et al., 2009; Budke and Goffinet, 2016). Contrasts in genic expression levels suggest also that differential length of the seta and the gain/loss of a specific opening mechanism can be achieved relatively easily by heterochronic expression of major developmental regulators, and hence that sporophyte reduction can occur by temporal and/or spatial reprogramming of expression of conserved regulatory modules. Whether convergent evolution of reduced sporophyte morphologies is driven by similar molecular mechanisms across unrelated moss lineages must await similar comparative studies from independent lineages. Ultimately, once the critical genetic traits underlying reduction are identified, the hypothesis on their irreversibility/reversibility can be tested. Together these advances would be essential to further our understanding of trends in the diversification of the moss sporophyte, and their integration in systematic concepts reflecting the relationships among mosses with highly contrasted morphologies.

## Data Availability Statement

RNA-seq data generated for this study have been submitted to the European Nucleotide Archive (ENA) under study accession number PRJEB36328. The draft genome sequence of *F. hygrometrica*, assembled transcripts and their protein translations are provided on figshare (https://doi.org/10.6084/m9.figshare.11663892.v1).

## Author Contributions

AK, PS, and BG conceptualized and designed the study. MW and ZB grew *F. hygrometrica* sporophytes and gametophytes, respectively, extracted RNA and prepared libraries. MR and AN collected *P. patens* sporophytes, extracted RNA, and prepared libraries. AK and PS analyzed the data and wrote the first version of the manuscript. All authors corrected and revised the final version of the manuscript.

## Conflict of Interest

The authors declare that the research was conducted in the absence of any commercial or financial relationships that could be construed as a potential conflict of interest.

## Supplementary Material

DATA SHEET S1 This data can be found at the link: https://doi.org/10.6084/m9.figshare.11663892.v1.
