# Supplementary material for: Transcriptional Landscapes of Divergent Sporophyte Development in Two Mosses, Physcomitrium (Physcomitrella) patens and Funaria hygrometrica
Source: Front Plant Sci. 2020 Jun 10;11:747. doi: 10.3389/fpls.2020.00747 (PMC7299128; doi:10.3389/fpls.2020.00747)
Supplement: Supplementary file 2 [file Table_1.DOCX]

Supplementary Material

# Supplementary Data

**Supplementary Data 1** Draft genome assembly, annotation, transcript sequences, and peptide sequences of *F. hygrometrica.*

**Supplementary Data 2** Results of differential expression analysis between gametophyte and sporophyte stages of *F. hygrometrica*, obtained by the DESeq2 pipeline. baseMean - mean of normalized counts over all samples; log2FoldChange - log_2_ fold-change sporophyte vs. gametophyte samples; lfcSE - standard error of log_2_ fold-change; stat - Wald statistic (log2FoldChange/lfcSE); pvalue - p-value for Wald statistic; padj - p-value adjusted for multiple testing.

**Supplementary Data 3** Results of differential expression analysis between gametophyte and sporophyte stages of *P. patens*, obtained by the DESeq2 pipeline. For header description see Supplementary Data 2.

**Supplementary Data 4** List of orthogroups (Orthofinder2) and orthologs between *F. hygrometrica* and *P. patens*.

**Supplementary Data 5** Significantly (p <= 0.05) enriched GO-terms of orthologs, preferentially expressed in the sporophyte stage of *F. hygrometrica.* GO.ID - identification number of assigned GO term; Term - description of assigned GO term; Annotated - number of genes with the assigned GO term in the gene universe; Significant - number of genes with the assigned GO term in the subset of preferentially sporophyte expressed genes; Expected - expected number of genes with the assigned GO term in the subset of preferentially sporophyte expressed genes; weightFisher - p-value for Fisher’s exact test statistic.

**Supplementary Data 6** Significantly (p <= 0.05) enriched GO-terms of non-orthologs, preferentially expressed in the sporophyte stage of *F. hygrometrica.* For header description see Supplementary Data 5.

**Supplementary Data 7** Significantly (p <= 0.05) enriched GO-terms of orthologs, preferentially expressed in the sporophyte stage of *P. patens.* For header description see Supplementary Data 5.

**Supplementary Data 8** Significantly (p <= 0.05) enriched GO-terms of non-orthologs, preferentially expressed in the sporophyte stage of *P. patens*. For header description see Supplementary Data 5.

**Supplementary Data 9** Assignment of orthologs between *F. hygrometrica* and *P. patens* to clusters and major clusters obtained by fuzzy c-mean clustering. ID - gene identifier; core.mem - cluster membership value; core - assigned cluster; DevStage - broad developmental stage at which expression peak of the cluster occurs, corresponding to major clusters 1-3 in Figure 5; Fh - *Funaria hygrometrica*; Pp - *Physcomitrella patens*.

**Supplementary Data 10** Orthologous transcription factor encoding genes assigned to minor and major clusters obtained by fuzzy c-means clustering. For header description see Supplementary Data 9; Family - transcription factor family the corresponding genes were assigned to.

# Supplementary Tables

**Supplementary Table 1** Read mapping statistics for RNAseq samples of *F. hygrometrica* and *P. patens*.

**
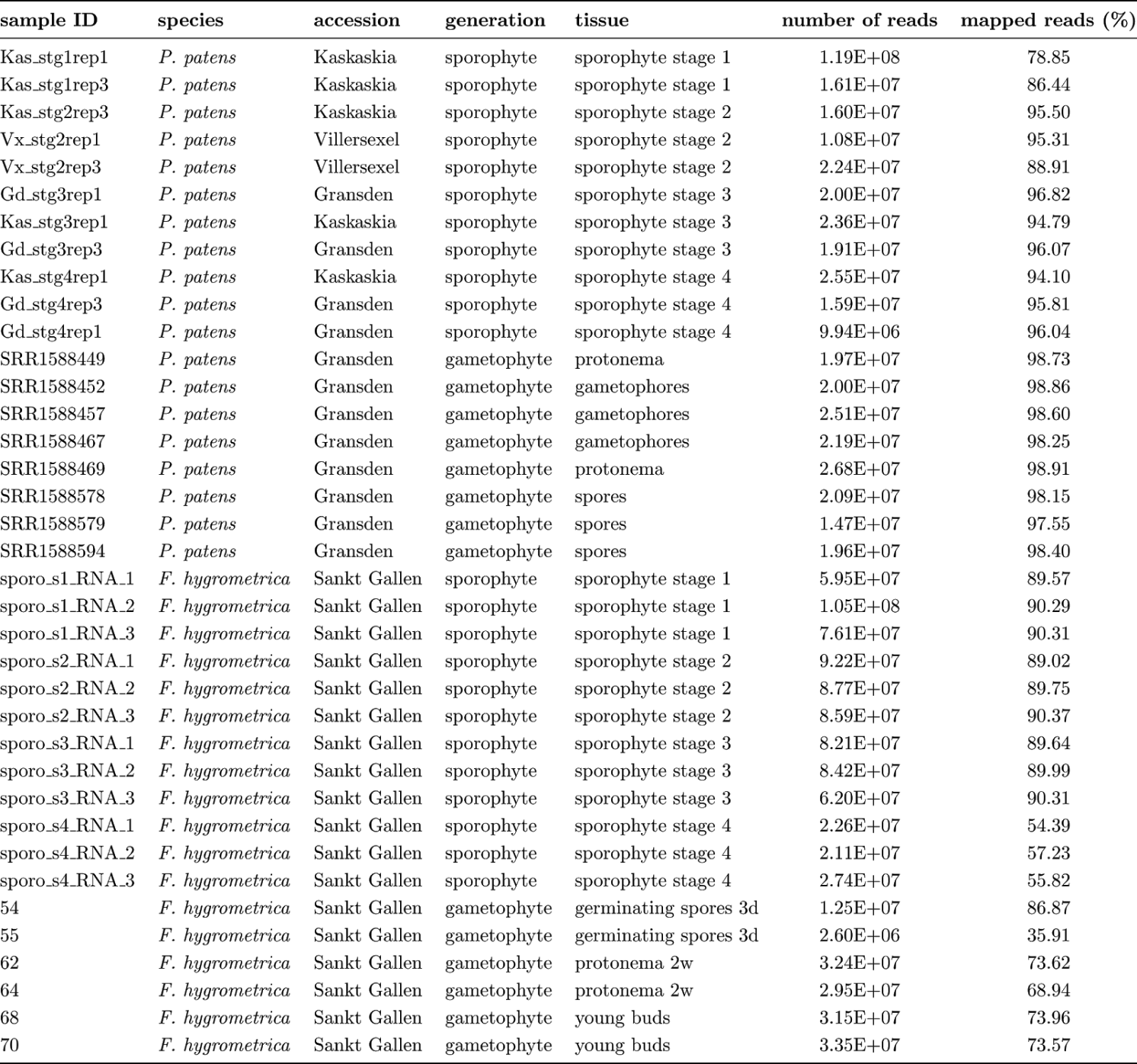
**
